# Structure-based prediction of SARS-CoV-2 variant properties using machine learning on mutational neighborhoods

**DOI:** 10.3389/fbinf.2025.1634111

**Published:** 2025-09-08

**Authors:** Max van den Boom, Erik Schultes, Thomas Hankemeier

**Affiliations:** 1 Department of Computer Science, Vrije Universiteit Amsterdam, Amsterdam, Netherlands; 2 Metabolics and Analytics Centre, Leiden Academic Centre for Drug Research (LACDR), Leiden University, Leiden, Netherlands; 3 GO FAIR Foundation, Leiden, Netherlands

**Keywords:** SARS-CoV-2, spike protein, receptor-binding domain (RBD), protein structure prediction, AlphaFold2, ESMFold, deep mutational scanning, variant of concern (VOC)

## Abstract

This dataset presents a structure-enriched resource of theoretical and empirical SARS-CoV-2 spike receptor-binding domain (RBD) variants, developed under the STAYAHEAD project for pandemic preparedness. It integrates large-scale *in silico* structure predictions with empirical biophysical measurements. The dataset includes 3,705 single-point Wuhan-Hu-1 RBD variants and 100 higher-order Omicron BA.1/BA.2 variants, annotated with AlphaFold2 and ESMFold metrics and Bio2Byte sequence-based predictors. Structural descriptors—RMSD, TM-score, plDDT, solvent accessibility, hydrophobicity, aggregation propensity—are linked to ACE2 binding and expression data from deep mutational scanning. Provided as a FAIR^2^ Data Package, it supports structure–function analysis, variant modeling, and responsible reuse in virology, structural biology, and computational protein science. This collaboration was co-funded by the PPP Allowance from Health ∼ Holland, Top Sector Life Sciences and Health, to stimulate public–private partnerships.

## 1 Introduction

The rapid emergence and global spread of SARS-CoV-2 variants during the COVID-19 pandemic underscored the urgent need for timely characterization of mutations in viral proteins (Korber et al., 2020; World Health Organization, 2023). Among these, the receptor-binding domain (RBD) of the spike (S) glycoprotein has been of particular interest due to its direct role in mediating viral entry via the human ACE2 receptor (Lan et al., 2020). Mutations in the RBD can significantly affect viral infectivity, immune escape, and pathogenicity (Harvey et al., 2021; McCallum et al., 2021), as observed in multiple Variants of Concern (VoCs) over the course of the pandemic. While genomic surveillance efforts have cataloged a vast diversity of spike variants, predictive frameworks capable of linking sequence variation to functional and biophysical consequences remain limited (McCallum et al., 2021; Starr et al., 2020).

This dataset was developed as part of the STAYAHEAD initiative, which investigates structure-informed approaches to forecasting the functional properties of emerging SARS-CoV-2 variants. The resource centers on the RBD of the ancestral Wuhan-Hu-1 spike protein and includes a systematically generated set of all possible single-point missense variants (3,705 in total), together with representative higher-order variants from the Omicron BA.1 and BA.2 lineages. These higher-order variants consist of full sets of mutations observed in actual clinical isolates of BA.1 and BA.2, typically involving 15–16 amino acid changes within the RBD. By including these real-world, multi-mutant sequences—rather than exhaustively enumerating all possible combinations—the dataset enables external validation of structure–function relationships derived from the 1-step variant analysis. These Omicron variants serve as extrapolation benchmarks to assess whether models trained on single-point mutations can generalize to more complex, naturally occurring mutational constellations. All variants are annotated with structural descriptors derived from two leading protein structure prediction tools—AlphaFold2 (Jumper et al., 2021) and ESMFold (Lin et al., 2023)—and augmented with sequence-based biophysical predictors such as disorder, flexibility, and aggregation propensity from the Bio2Byte suite (Cilia et al., 2013).

To complement structural predictions, empirical measurements of ACE2 binding affinity and surface expression levels were integrated for each 1-step variant, based on deep mutational scanning (DMS) data published by Starr et al. (Starr et al., 2020). This pairing of theoretical and experimental annotations provides a foundation for analyzing structure–function relationships across the spike mutational landscape and supports downstream applications in protein modeling, variant characterization, and structure-guided surveillance.

This article presents a detailed description of the FAIR (Cilia et al., 2013) dataset, including the methods used for its generation, the scope of structural and functional annotations, and its potential uses in virology, structural biology, and pandemic preparedness. The dataset is made available as a FAIR^2^-compliant package, designed to reduce ambiguity and enrich contextual metadata, while supporting traceability and reproducibility across analytical and modeling workflows.

## 2 Methods summary

This methods summary details the design, generation, and preparation of a structured, AI-ready dataset linking SARS-CoV-2 spike protein variants to biophysical properties relevant to viral fitness. The dataset was produced within the framework of Project STAYAHEAD, a pandemic preparedness initiative, and is fully documented in the accompanying FAIR^2^ Data Package and Portal. It includes theoretical variant modeling, large-scale structure prediction, quantitative feature extraction, integration of empirical data, and the construction of machine learning–ready matrices. All steps were conducted with an emphasis on reproducibility, modular processing, and alignment with FAIR principles, as well as AI-Readiness and Responsible AI.

### 2.1 Study design

The methodological framework was developed to assess whether predicted structural features of SARS-CoV-2 spike protein variants could be used to anticipate empirical properties associated with increased viral transmissibility and immune evasion. The project focused specifically on the ACE2 receptor-binding domain (RBD) of the spike protein, which comprises 195 amino acids and plays a key role in viral entry into host cells. This subdomain was selected through expert consultation with virologists at Utrecht University, based on its relevance to receptor binding and its known accumulation of mutations across variants of concern (VoCs). The reduced sequence length of the RBD also made it tractable for high-throughput structural modeling.

To enable systematic exploration of mutational effects, the sequence space was defined using a k-step substitution framework. The 1-step neighborhood, consisting of all single amino acid substitutions, generated 3,705 unique variants. The 2-step neighborhood expanded this to over 6.8 million theoretical double mutants. These variants were conceptualized as concentric rings of increasing mutational distance around the reference RBD sequence from Wuhan-Hu-1 (PDB: 6M0J). While the full space was mapped, this study focused primarily on the 1-step variants for model training and used higher-order variants for future extrapolation testing.

To ensure transparency and consistency, the dataset was organized into five curated sequence subsets: (1) a scalability set of 72 synthetic sequences spanning lengths from 10 to 1,273 residues; (2) a validation pair consisting of the full-length 1,273-residue spike protein and the 195-residue RBD; (3) the 1-step and 2-step mutational neighborhoods; (4) empirically observed VoCs including Alpha through Omicron lineages; and (5) 890 control spike glycoproteins with experimentally determined structures (via X-ray or Cryo-EM), selected from the Protein Data Bank. These subsets served different roles in benchmarking, domain validation, and performance assessment.

### 2.2 Variance sequence generation

The complete theoretical variant space was generated computationally using a custom Python script, ViralMutations.py, designed to introduce amino acid substitutions into the RBD sequence provided in FASTA format. The script supported exhaustive generation of k-step mutational neighborhoods and permitted the construction of both complete and selective variant subsets. For 1-step variants, each position in the reference sequence was substituted with all 19 alternative residues, yielding 3,705 unique sequences. The 2-step set was generated by introducing two non-repeating substitutions per sequence, resulting in 6,828,315 combinations.

Each variant was saved as an individual FASTA record, labeled with a unique identifier that captured both the position and the identity of the mutation (e.g., N501Y). The generated sequences were grouped into logical subsets for downstream processing. The scalability subset included 16 sequence lengths sampled from the full spike protein, each represented by five randomly selected segments. The paired validation subset contained the full-length spike and the isolated RBD for comparative modeling. The observed variant subset included RBD sequences derived from clinical isolates spanning multiple VoCs. Control sequences were obtained from the Protein Data Bank and filtered by length (≤1,278 residues) and structural quality (verified by X-ray crystallography or Cryo-EM). All sequences were checked for formatting compliance and naming consistency before passing into the modeling pipeline.

### 2.3 Protein structure prediction

Three structure prediction tools were used: AlphaFold-2 (AF2), ESMFold, and AlphaFold-Pulldown (AF-PD). All predictions were executed within a distributed infrastructure consisting of the Snellius Supercomputer (SURF), a local development workstation, and a shared 2 TB Research Drive managed via OwnCloud. Prediction scripts were tested interactively on Snellius using SBATCH and then submitted for batch processing.

AlphaFold-2 (version 2.3.1) was deployed using the full required reference databases: BFD, MGnify, PDB70, UniRef30, UniRef90, and the full PDB, totaling approximately 2.62 TB. Multiple sequence alignments (MSAs) were constructed using HHblits and JackHMMer, and structural templates were retrieved via HHSearch. AF2 generated five ranked models per sequence, along with MSA files, template alignments, and a serialized features. pkl file containing residue-level input features and confidence metrics. All outputs were stored in PDB format and labeled with a sequence identifier, prediction rank, and timestamp.

ESMFold was used for high-speed, template-free prediction of single-chain structures. It utilized a pre-trained 15-billion parameter language model (ESM-2) and required only the input sequence in FASTA format. The model generated a single deterministic prediction per sequence, significantly reducing computational overhead compared to AF2. It did not generate alignments or templates but provided comparable accuracy for many sequences.

AlphaFold-Pulldown (AF-PD) was used to model RBD–ACE2 complexes. The tool extended AF2-Multimer functionality and accepted the features. pkl files from AF2 as input. The “pulldown” mode allowed high-throughput prediction of protein-protein interactions. Output files included complex PDBs, interfacial contact maps, per-residue confidence scores, and interaction metrics such as pDockQ, mpDockQ, ipTM, and PI-Score. Computational tasks were distributed across CPU and GPU partitions to maximize throughput.

File sizes ranged from 124 KB per sequence for ESMFold predictions, to 97.1 MB for AF2 outputs, and up to 1.6 GB for AF-PD complexes. All files were hash-verified and archived under versioned directories by variant subset and tool.

### 2.4 Structural feature extraction

Each predicted structure was analyzed to extract a range of structural, surface, and interaction features using open-source software and domain-specific pipelines. Global similarity to the reference structure (PDB: 6M0J) was assessed using RMSD and TM-score, calculated via BioPython (Kabsch algorithm) and TM-align (tmtools). SASA was calculated using FreeSASA with both Lee-Richards and Shrake-Rupley implementations. Atom radii were assigned via the ProtOr scale. Electrostatic potentials were computed using APBS after PDB2PQR conversion and run with the PARSE force field.

AlphaFold confidence scores (pLDDT, pTM, ipTM) were parsed from the original prediction outputs, while ESMFold only contains a single confidence score (pLDDT). AF-PD scores were extracted directly from the result_model_x.pkl files and included interface-specific metrics such as pDockQ, mpDockQ, iPAE, and the PI-Score (an ML-based score trained on Cryo-EM assemblies). Additional interaction metrics such as hydrogen bonds, salt bridges, shape complementarity, and solvation energy were computed from the predicted interfaces.

To complement structure-derived features, per-residue predictive features were computed using the Bio2Byte b2btools package. These included backbone flexibility (DynaMine), early folding propensity (EFoldMine), disorder (DisoMine), and amyloid aggregation tendency (AgMata). Each per-residue feature was averaged across the sequence to yield a single value per variant. All features were compiled into tabular format and indexed by sequence ID.

### 2.5 Integration of empirical data

Empirical biophysical data were sourced from deep mutational scanning experiments conducted by the BloomLab, covering 1-step ACE2–RBD variants. Two metrics were used: binding affinity (log (KD)) and surface expression (log (MFI)), measured using ACE2 binding assays and flow cytometry, respectively. Wild-type reference values were used to calculate Δ (delta) values, capturing the relative effect of each mutation on binding or expression.

Variant identifiers were matched via mutation labels (e.g., N501Y), and a mapping table was created to align each structure with its empirical measurement. Variants lacking empirical data were excluded. The final matched dataset included 3,705 variants with complete empirical and structural records. Target variables were appended to the feature table, and all mappings were verified for consistency.

### 2.6 Machine learning dataset construction

To support downstream modeling, the complete dataset was standardized, partitioned, and saved in multiple formats. Structural features were min-max normalized using StandardScaler from scikit-learn, while target variables were left untransformed. Datasets were split into training (80%) and holdout (20%) sets using train_test_split. Five-fold cross-validation was implemented within the training set. Two target configurations were supported: one with binding and expression as targets, and another including delta binding and delta expression.

Each record in the final matrix included a unique sequence ID, structural features, and empirical targets. Datasets were saved as. csv and. pkl files with associated metadata descriptors and schema. All versions were archived with identifiers for variant subset, model type, and training configuration. A complete record of the dataset construction, including preprocessing scripts and provenance logs, is available through the FAIR^2^ Data Portal.

## 3 Data overview

### 3.1 Data summary

This dataset compiles structural and empirical data on SARS-CoV-2 spike receptor-binding domain (RBD) variants, generated within the STAYAHEAD project to support AI-driven pandemic preparedness. Focusing on the 195-residue ACE2–RBD (PDB: 6M0J), it includes both clinically observed and theoretically generated missense variants. Structural features were predicted using AlphaFold2 (AF), AlphaFold-Pulldown (AF-PD), and ESMFold (ESM), and were enriched with biophysical descriptors from the Bio2Byte toolkit. Empirical ACE2 binding and RBD expression values from deep mutational scanning serve as training targets for machine learning models.

The dataset comprises five defined subsets—including mutational scans, clinical isolates, benchmarking sequences, and experimentally resolved controls—and is formatted for FAIR^2^ compliance, machine learning readiness, and reproducibility. cosystem management.

In line with FAIR^2^ documentation practices, the term “resources” is used to refer to datasets, files, and other digital assets contained within the data package. This naming convention reflects common usage across FAIR-aligned platforms and supports consistency with the portal structure. The full list of named datasets—including their concise, machine-actionable filenames is available and indexed in the Resources section of the FAIR^2^ Data Portal [https://www.doi.org/10.71728/hw56-vj34]. This ensures accurate traceability and avoids redundancy between the manuscript and data portal, which together form an integrated data publication.

### 3.2 Quantitative summary of the dataset

This section describes the dataset’s composition, structure prediction metrics, feature annotations, model performance, and associated computational costs.

#### 3.2.1 Dataset composition

The complete dataset includes a total of 6,833,011 protein sequences, grouped across five major categories:

- 1-step variants (n = 3,705): All possible single-residue missense mutations of the RBD reference sequence.
- 2-step variants (n = 6,828,315): Exhaustive enumeration of double mutations for combinatorial modeling.
- Observed RBD variants (n = 67): Clinical isolates from documented SARS-CoV-2 lineages.
- Benchmark and control sequences (n = 964): Including 890 structurally verified spike proteins from PDB and 72 sequences for benchmarking model runtime and scalability.
- Validation variants (n = 100 each for BA.1 and BA.2): Higher-order Omicron variants used to assess model generalization across 15–16 mutation steps.

#### 3.2.2 Structure prediction performance

Protein structures were predicted using three tools with differing algorithmic properties:

- AlphaFold2 (AF v2.3.1) yielded highly accurate structures for wild-type and low-mutation variants:
  - RMSD: 0.63 ± 0.02 Å
  - TM-score: 0.97 ± 0.001
  - Output size: ∼97 MB per structure
- AlphaFold-Pulldown (AF-PD) provided protein–protein interface metrics, targeting ACE2–RBD interactions:
  - RMSD: 0.69 ± 0.02 Å
  - TM-score: 0.91 ± 0.001
  - Output size: ∼1.6 GB per structure
- ESMFold offered fast, alignment-free structure prediction, trading off accuracy for scalability:
  - RMSD: 19.28 Å
  - TM-score: 0.28
  - Output size: ∼124 KB per structure

#### 3.2.3 Annotated feature space

For each variant, a set of structural and sequence-based features was computed:

- Structural descriptors: RMSD, TM-score, plDDT, solvent-accessible surface area (SASA), and electrostatic potential.
- Multimeric interaction scores (AF-PD only): pDockQ, mpDockQ, interface pLDDT, iPAE, PI-score, binding energy.
- Sequence-derived features (Bio2Byte): AgMata (amyloid aggregation), DisoMine (disorder), EFoldMine (early folding), DynaMine (flexibility).

These features form a structured input space for machine learning models, with 15–20 numeric descriptors per sequence.

#### 3.2.4 Model performance

Model performance was quantified for both binding and expression prediction tasks:

- Best model (expression):
  - RMSE: 0.63
  - R^2^: 0.42
- Best model (binding):
  - RMSE: 0.86
  - R^2^: 0.34
- Top-ranked features across all models included AgMata, plDDT, RMSD, EarlyFolding, and TM-score.

#### 3.2.5 Predictive generalization

Models were validated on 100 Omicron BA.1 and 100 BA.2 variants, each containing 15–16 mutations. ESM-based models achieved high predictive fidelity, with three out of four mean predicted binding and expression values falling within one standard deviation of empirical measurements, supporting generalization beyond 1-step variants.

### 3.3 FAIR^2^ compliance certification

The dataset supporting the findings of this study is available through a FAIR^2^ Data Portal (https://www.doi.org/10.71728/hw56-vj34), which ensures that the data adhere to the principles of Findability, Accessibility, Interoperability, and Reusability (FAIR), with additional emphasis on including detailed Contextual metadata and AI-Readiness and Responsible AI practices (Wilkinson et al., 2016). All raw data, metadata, and supplementary materials, including detailed protocols and methods, are accessible via the FAIR^2^ Data Portal https://www.doi.org/10.71728/hw56-vj34.

The dataset has been structured to ensure compliance with FAIR^2^ standards, enabling easy integration with other datasets and promoting reuse in future research (Table 1). Researchers can access the dataset in multiple formats, and appropriate documentation is provided to facilitate transparency and reproducibility. Any updates or corrections to the dataset will also be managed and tracked through the portal, ensuring long-term accessibility and version control.

**TABLE 1:** The FAIR^2^ Compliance Certification presented here was generated through a Human-in-the-Loop (HITL) process combining automated FAIR^2^ system checks with author-supplied inputs. While certain metadata fields and validations (e.g., DOI registration, schema adherence, file accessibility) are verified automatically by the FAIR^2^ platform, other elements—such as domain-specific documentation quality and Responsible AI considerations—reflect expert curation by the dataset authors.

Criteria	Assessment	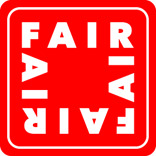
Findability (F)		
**F1. Unique identifier**	The dataset is assigned a globally unique and persistent DOI (https://www.doi.org/10.71728/hw56-vj34), ensuring it can be reliably cited.	
**F2. Metadata**	Metadata includes key fields such as title, creator(s), description, keywords, and versioning. Key variables include SARS-CoV-2, Spike protein, RBD, AlphaFold2, ESMFold.	
**F3. Metadata includes data identifiers**	The metadata explicitly references the dataset’s DOI, ensuring strong linkage between metadata and the data package.	
**F4. Searchable metadata**	Controlled vocabularies from schema.org and MLCommons Croissant are used for key properties. CRediT taxonomy captures contributor roles.	
**Indexed in repositories**	Indexed in the FAIR² Data Portal and DataCite. Registration with general-purpose repositories (e.g., Zenodo) would broaden accessibility.	
Accessibility (A)		
**A1. Open access**	The dataset is openly accessible without restriction, in alignment with open science practices.	
**A2. Long-term access**	Archival is managed within the FAIR² Data Package, which includes long-term access provisions.	
**Package and metadata access**	Access to the dataset is provided via DOI-based redirection, which resolves to a FAIR²-compliant metadata record and downloadable data package.	
Interoperability (I)		
**I1. Standardized formats**	Data is available in CSV and JSON formats, compatible with common data science and bioinformatics platforms. Croissant schema enhances interoperability.	
**I2. Controlled vocabularies**	Key descriptors follow schema.org, MLCommons, and CRediT taxonomies. Domain-specific ontologies could further strengthen cross-dataset integration.	
**I3. Cross-platform integration**	The dataset aligns with best practices for biomedical and AI-ready datasets. It can be integrated with machine learning, visualization, and modeling pipelines.	
Reusability (R)		
**R1. Comprehensive documentation**	Accompanied by extensive documentation, including data dictionaries, methods, and preprocessing steps.	
**R1.1. License**	Licensed under ODC-By v1.0, permitting reuse, redistribution, and modification with attribution.	
**R1.2. Detailed provenance**	Metadata includes origin of all data sources, author contributions (via CRediT), and transformation history. Preprocessing and structural prediction methods are described using the PROV-O ontology.	
**R1.3. Domain-relevant standards**	Dataset is aligned with MLCommons Croissant for AI/ML datasets. No domain-specific biomedical standard (e.g., MIAME) was aplicable.	
**Versioning and updates**	Dataset includes version metadata. Future updates will include changelogs and semantic versioning.	
AI-Readiness (AIR)		
**Structured for machine learning**	Data is clearly labeled with categorical and numerical types, standardized column names, and consistent formatting suitable for model pipelines	
**Scalable**	Files are optimized for batch processing and large-scale variant screening. Data can be processed using HPC or cloud-based systems.	
**Training and validation sets**	Partitioning by mutation class (e.g., 1-step vs. Omicron BA.1/BA.2) enables robust training/validation splits for supervised learning tasks.	
Responsible-AI (RAI)		
**Ethical standards and misuse**	The dataset is intended for biological modeling and screening; no personally identifiable data included.	
**Biases in the dataset**	Reflects mutation coverage and structure prediction tool availability; no demographic, social, or geographic data are present.	
**Data privacy and security**	Contains no personally identifiable information. Data integrity is ensured through static archiving; no encryption needed.	
**Fairness and non-discrimination**	Neutral scientific dataset. Appropriate for objective benchmarking and exploratory model development.	
**Explainability and interpretability**	Variables are documented with clear definitions and units. Input features used in machine learning are described, including transformations.	
**Data provenance and accountability**	Well-described fields support interpretability. Additional documentation could enhance transparency of transformation steps.	
**Transparency and reporting**	Dataset includes traceability metadata.	
**Ethical and social impact**	The dataset’s relevance to environmental policy and conservation highlights its societal impact, though further ethical guidance on use cases (e.g., in ecosystem management) could support users applying the data in high-stakes contexts.	
**Human-in-the-loop (HITL) considerations**	Can be used to support HITL scenarios in structure-function prediction pipelines or in hypothesis generation tools.	

#### 3.3.1 Overall FAIR^2^ badge compliance

**Compliant**–The dataset qualifies for the FAIR^2^ Badge, meeting all requirements across Findability, Accessibility, Interoperability, Reusability, AI-Readiness, and Responsible AI. Suggested enhancements include more detailed metadata on data transformations and validation, clearer descriptions of sampling biases, and ethical guidance for specific AI applications.

## 4 Visual overview

To support intuitive understanding of the dataset’s structure and content, we provide a set of summary visualizations that highlight key aspects of dataset size, feature distribution, and inter-variable relationships.

**FIGURE 1:**
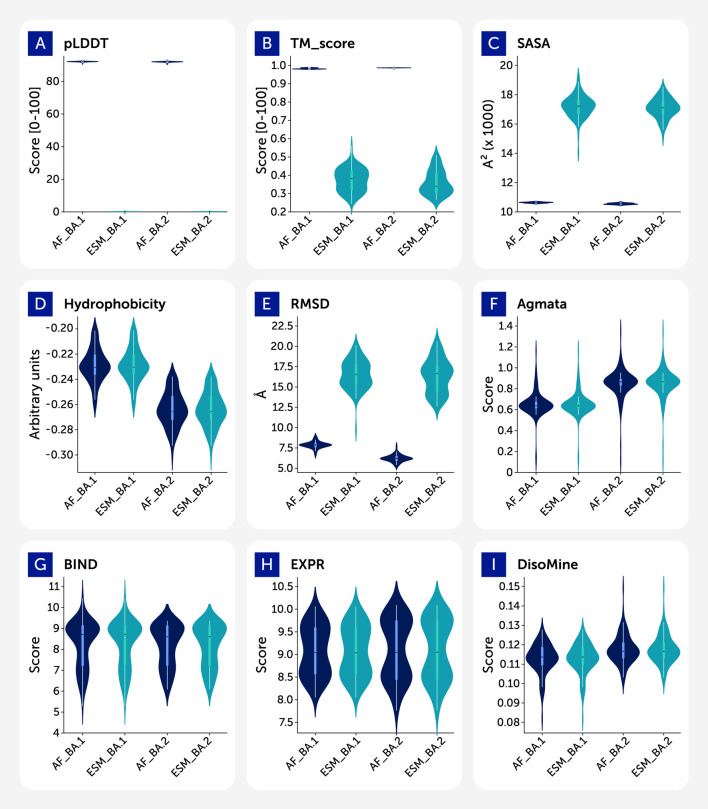
Violin plots of feature distributions by dataset.

Figure 1 reveals distinct differences in the distribution of core features. RMSD and plDDT show a clear separation between AlphaFold2 and ESMFold predictions, with ESMFold producing lower-confidence and more variable structures. TM-score distributions are tightly peaked for AF datasets, while flatter and lower for ESMFold. Amyloid aggregation propensity (AgMata) and disorder (DisoMine) features exhibit consistent patterns across tools, but subtle shifts in BA.1 and BA.2 suggest functional divergence. The empirical variables (ACE2 binding and RBD expression) show greater spread in the 1-step variants and higher central values for Omicron variants.

**FIGURE 2:**
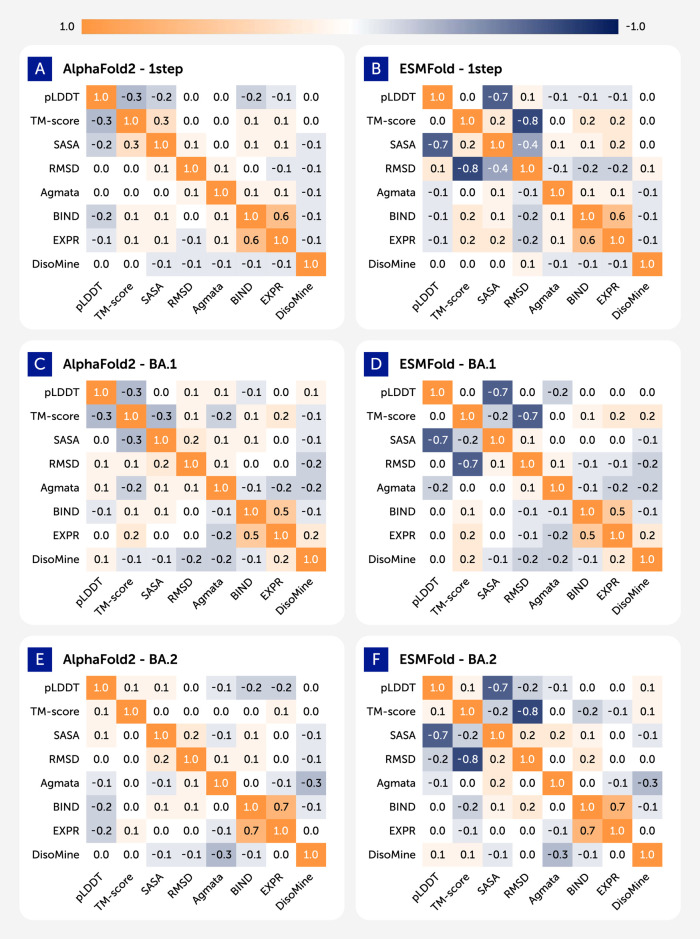
Heatmaps showing pairwise pearson correlations between structural and biophysical features across different datasets. **(A)** AlphaFols2 - 1step. **(B)** ESMFold - 1step. **(C)** AlphaFols2 - BA.1. **(D)** ESMFold - BA.1. **(E)** AlphaFols2 - BA.2. **(F)** ESMFold - BA.2.

Figure 2 presents the Pearson correlation matrices illustrating the relationships between key biophysical properties of the spike receptor-binding domain (RBD). The analysis covers three distinct variant datasets: the ancestral Wuhan-Hu-1 (labeled as “1-step”), Omicron BA.1, and Omicron BA.2. The matrices were generated independently for predictions from AlphaFold2 (left column) and ESMFold (right column). To ensure comparability, the analysis uses a consistent set of five features available across all datasets: the predicted LDDT score (pLDDT), the template modeling score (TM-score), the solvent-accessible surface area (SASA), relative Hydrophobicity, and the root-mean-square deviation (RMSD).

The resulting heatmaps display distinct correlation patterns that vary by prediction method and viral lineage. A consistently strong positive correlation (r > 0.8) is evident between pLDDT and TM-score in all conditions, which aligns with their function as metrics of model confidence and structural accuracy. In contrast, relationships between other structural metrics and model confidence scores show notable variability. For example, the correlation between RMSD and pLDDT is moderately negative in the AlphaFold2 predictions for all variants (Panels A, C, E), whereas this correlation is substantially weaker or near-zero in the corresponding ESMFold predictions (Panels B, D, F). The figure provides a comparative overview of the statistical interplay between predicted structural features, documenting the differing outputs of the two computational models.

**FIGURE 3:**
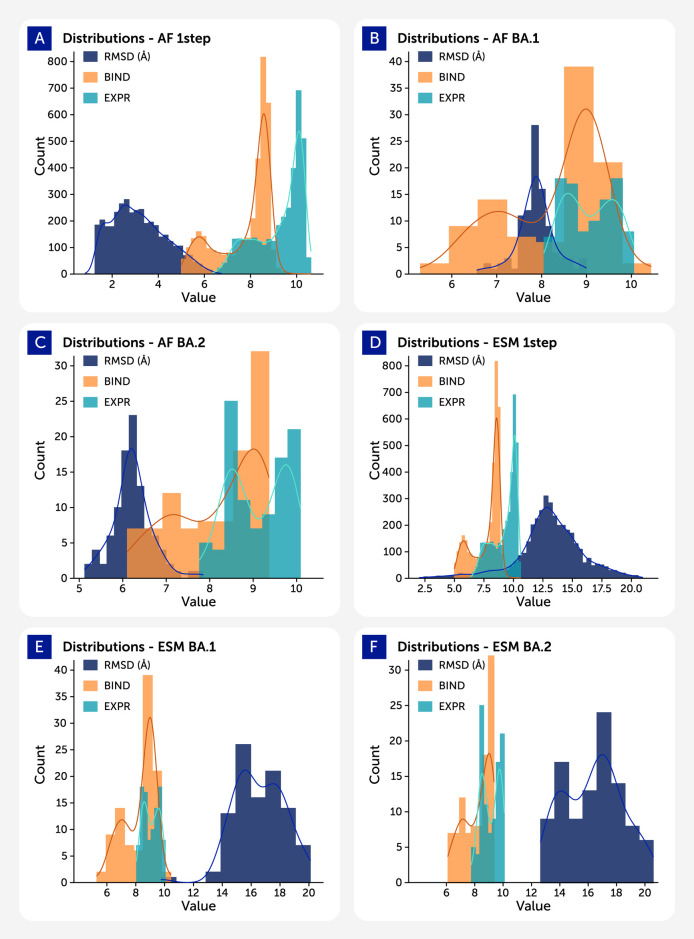
Histograms depicting the distributions of RMSD (blue), binding affinity (log KD, red), and expression levels (log MFI, green) across different datasets. Datasets generated using AlphaFold2 are labeled with the prefix “AF_”, while those generated with ESMFold use the prefix “ESM_”.

In Figure 3, each panel shows overlaid distributions of RMSD (blue), ACE2 binding affinity (log KD, red), and RBD surface expression (log MFI, green) for different datasets generated using either AlphaFold2 (AF) or ESMFold (ESM). The left column displays AlphaFold2-based predictions: AF_1step (top), AF_BA2 (middle), and AF_BA1 (bottom), while the right column shows ESMFold-based datasets: ESM_1step (top), ESM_BA2 (middle), and ESM_BA1 (bottom).

RMSD distributions (blue) highlight model-dependent differences in structural confidence. AlphaFold2-predicted structures are tightly centered around lower RMSD values (∼3–6 Å), reflecting higher structural consistency, particularly in AF_1step. In contrast, ESMFold predictions show broader and more variable RMSD distributions (typically ∼10–16 Å), indicating less precise structural outputs.

Binding affinity (red) distributions for 1-step datasets cluster near the wild-type range (KD ∼8–9), while those for BA.1 and BA.2 variants shift slightly lower, suggesting reduced ACE2 interaction in many Omicron variants. Expression values (green) show similar mutational sensitivity, with altered distributions in BA.1 and BA.2 datasets—especially in AF_BA1 and ESM_BA2—potentially reflecting impacts on folding or stability.

Together, these plots summarize how variant class and prediction model influence structure quality and predicted biophysical function. Clear trends in RMSD distinguish AF2 vs ESMFold, while changes in binding and expression highlight mutational effects across variant groups.

## 5 Discussion

### 5.1 The value of the dataset

This dataset provides a structured, extensible, and richly annotated resource for analyzing how amino acid mutations in the SARS-CoV-2 spike receptor-binding domain (RBD) affect protein structure and viral function. Its strength lies in combining three complementary dimensions: (1) large-scale theoretical mutational coverage via single-residue substitutions, (2) high-quality predicted structural features from two distinct structure prediction tools, and (3) experimental data derived from deep mutational scanning.

By systematically enumerating and structurally characterizing all 1-step missense variants from the Wuhan-Hu-1 RBD, the dataset enables fine-grained analysis of mutational landscapes in a biologically critical region of the spike protein. The inclusion of real-world, higher-order variants from Omicron lineages (BA.1 and BA.2) allows validation of structure-function relationships in emerging variants, and supports generalization of computational findings.

The dual use of AlphaFold2 and ESMFold provides insight into tool-specific biases and variability in structure prediction. AlphaFold2 offers high-confidence, template-based structures, while ESMFold captures sequence-based generalization without reliance on templates. The addition of Bio2Byte-derived sequence features, such as aggregation propensity and disorder, further enriches the representation of molecular properties and increases compatibility with machine learning workflows.

This dataset bridges a critical gap between sequence surveillance and functional interpretation. It provides the foundational features needed to model, classify, or rank novel spike variants by their potential impact on ACE2 binding and RBD expression—two properties closely associated with infectivity and immune evasion. Its structure also supports benchmarking and training of predictive algorithms, particularly for datasets with similar dimensionality and biophysical complexity.

Because it is published as a FAIR^2^ Data Package, this resource emphasizes not only content completeness but also accessibility, provenance, and reusability. Detailed metadata, transparent data provenance, and standardized annotations ensure that the dataset is readily reusable across structural bioinformatics, computational virology, and pandemic preparedness applications.

### 5.2 The limitations of the dataset

Despite its comprehensive scope and careful design, this dataset has several limitations that should be acknowledged for responsible reuse and interpretation.

First, the dataset is centered on the ACE2 receptor-binding domain (RBD) of the SARS-CoV-2 spike protein, comprising 195 residues from the original Wuhan-Hu-1 reference strain. While this domain is functionally critical and harbors the majority of high-impact mutations, it represents only a subset of the full spike protein (1,273 residues) and does not account for mutations outside the RBD that may affect spike trimerization, fusion dynamics, immune escape, or protein–protein interactions.

Second, the dataset focuses on single-point (1-step) mutational variants for model training. While this strategy ensures interpretability and exhaustive coverage, the ability to generalize to higher-order variants (e.g., BA.1 and BA.2) is constrained by the combinatorial complexity of multi-mutation effects, which may not be linearly additive. Epistatic interactions—where the effect of one mutation depends on the presence of others—are not explicitly modeled in the feature space, though some may be captured implicitly through empirical measurements.

Third, structure prediction outputs vary by tool and carry intrinsic confidence limitations. AlphaFold2 provides high-quality structures with reliable plDDT scores, but requires substantial computational resources and access to large alignment databases. ESMFold, while faster and alignment-free, produces lower-confidence structures with higher structural variability, particularly for longer and more mutated sequences. The dataset includes both tools to support comparative analysis, but users should interpret structure-based features in light of their respective confidence scores.

Fourth, although empirical binding and expression data are included for all 1-step variants and selected Omicron mutational variants, these measurements derive from deep mutational scanning in a controlled *in vitro* setting. They may not fully reflect the biophysical context of the full-length spike protein or *in vivo* viral dynamics. Additionally, the antibody escape measurements from the original source were excluded from this dataset, limiting its immediate applicability for immunogenicity modeling.

Finally, the dataset includes only variants derived from a single reference lineage (Wuhan-Hu-1) and does not capture natural diversity across global SARS-CoV-2 lineages or host-specific adaptations. As such, it should be treated as a mutational perturbation space rather than a direct reflection of global viral evolution.

These limitations do not undermine the dataset’s utility but highlight important boundaries for interpretation. Future expansions could incorporate additional mutational steps, full spike context, more diverse lineages, and complementary empirical assays to enhance biological realism and modeling capacity.

## 6 Conclusion

The dataset provides a structure- and function-annotated resource for studying the mutational landscape of the SARS-CoV-2 spike receptor-binding domain (RBD). It integrates comprehensive 1-step missense variants with higher-order Omicron mutational variants and combines structural predictions from AlphaFold2 and ESMFold with sequence-based biophysical features and deep mutational scanning data. The result is a curated, multi-modal dataset designed to facilitate the analysis of how amino acid substitutions influence spike protein conformation, receptor binding, and surface expression.

By systematically covering all single-residue RBD mutations and providing paired theoretical and empirical annotations, the dataset supports diverse applications, including structural modeling, functional prediction, mutational effect analysis, and variant risk prioritization. The inclusion of real-world mutational variants from the Omicron BA.1 and BA.2 lineages enables external validation of learned structure–function relationships, and the dual-model approach (AF2 and ESMFold) allows users to compare prediction strategies and confidence profiles.

Published as a FAIR^2^ Data Package, the dataset emphasizes not only scientific depth but also data stewardship. It ensures that variables are contextualized, provenance is transparent, and structure–function mappings are reproducible and traceable. This enables its responsible reuse across computational virology, protein bioinformatics, and emerging infectious disease preparedness.

While the dataset is focused on a specific region of the spike protein and has limitations related to generalizability and full-protein context, it provides a foundation for future expansions. These may include higher-order mutational combinations, extended domain coverage, and integration with immune and host interaction data.

In summary, this resource contributes to the broader effort of translating viral sequence data into mechanistic insight. It offers researchers and practitioners a flexible, well-annotated platform for investigating the molecular consequences of SARS-CoV-2 spike mutations in support of variant characterization and pandemic response strategies.

The dataset supporting the findings of this study is available through a FAIR^2^ Data Portal, which ensures that the data adhere to the principles of Findability, Accessibility, Interoperability, and Reusability (FAIR), with additional emphasis on including detailed Contextual metadata and AI-Readiness and Responsible AI practices. All raw data, metadata, and supplementary materials including detailed protocols and methods, are accessible via the FAIR^2^ Data Portal [https://www.doi.org/10.71728/hw56-vj34].

## Data Availability

The datasets presented in this study can be found in online repositories. The names of the repository/repositories and accession number(s) can be found in the article/supplementary material.
